# Bilateral Occipital Arteries of Internal Carotid Origin: Report of a Case and Review of the Literature

**DOI:** 10.5334/jbr-btr.828

**Published:** 2015-09-15

**Authors:** N. Çetin, K. Akkan, M. Uçar, B. Onal, E. Ilgit

**Affiliations:** 1 Department of Radiology, Gazi University School of Medicine, Ankara, Turkey

**Keywords:** Arteries, abnormalities – Arteries, stenosis or obstruction

## Abstract

The present report describes a patient with bilateral occipital arteries of internal carotid origin, which is an extremely rare variation, and left vertebral artery ostial stenosis diagnosed by selective catheterization and digital subtraction angiography.

The occipital artery is a main branch of the external carotid artery [1]. Anomalous origin of the occipital artery from internal carotid artery is an extremely rare variation [2,3,4,5,6,7,8,9,10,11,12,13]. Bilaterality of this variation is much more rare in the literature and there are only two cases reported previously [2,3,4,5,6,7,8,9,10,11,12,13]. The aim of this report is to explain the embriological origin of this variation, to review the literature, to emphasize its clinical importance, and also to contribute to the literature by presenting a case with bilateral form.

## Case report

**Figure 1:**
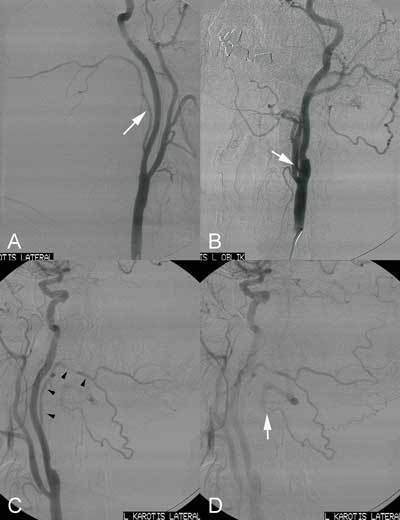
A. Selective right carotid arteriography in lateral projection showing anomalously originating right occipital artery from the posterior aspect of the internal carotid artery (arrow). B. Selective left carotid arteriography in lateral projection showing anomalously originating left occipital artery from the postero-medial aspect of the internal carotid artery (arrow). C, D. Its route (arrowheads) and anastomosis with left vertebral artery (arrow).

**Figure 2:**
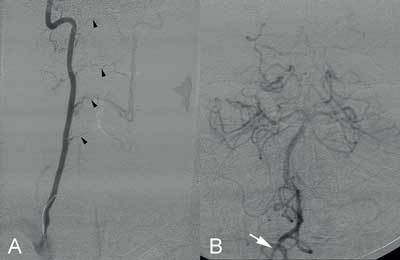
A. Right vertebral angiography demonstrating intervertebral collateral flow to the left vertebral artery (arrowheads). B. Hypoplasic V3–V4 segments of the right vertebral artery (arrow).

**Figure 3:**
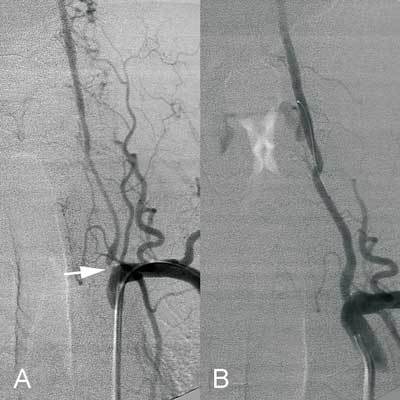
A. Left subclavian arteriography revealing high grade ostial stenosis of the left vertebral artery (arrow). B. Post-stent arteriography showing patency of the left vertebral artery with smooth contours and diminished collateral flow from the cervical arteries.

A 63-year-old man followed up for diabetes mellitus, hypertension and recently increasing vertigo with bilateral murmer at his neck. The patient had no neurological deficit and referred to radiology for the examination of cerebrovascular diseae. Bilateral carotid color duplex Doppler examination showed mild atherosclerotic plaques of the common carotid bifurcation without any hemodynamically significant stenosis. Cerebral MRI revealed chronic ischemic signal changes in pons and nonspesific signal changes in white mater of bilateral frontal lobes. Selective carotid and vertebral angiographies were performed in order to confirm the stenoses in proximal parts of both internal carotid arteries; occipital arteries were arising from the cervical segments of internal carotid arteries on both sides (Fig. 1). Selective right vertebral angiography revealed the patency of the artery with hypoplasia of V3–V4 segments and intracranial and intervertebral collateral flow to the left vertebral artery (Fig. 2). Left subclavian angiography prior to catheterization of vertebral artery demonstrated severe stenosis of the vertebral artery. Left vertebral artery is also opacified through the anastomoses between the muscular branches of occipital and vertebral arteries (Fig. 3A). A baloon-expandable intravascular stent was placed to the ostium of the left vertebral artery in order to treat the high grade stenosis (Fig. 3B).

## Discussion

Occipital artery arises from the posterior aspect of the external carotid artery, runs on the medial surface of the posterior belly of the digastric muscle and ends in the posterior part of the scalp [1]. However, occipital arteries of internal carotid origin have been reported as rare variants in the literature beginning from the anatomical studies in nineteenth century [2,3,4,5,6,7,8,9,10,11,12,13].

The first radiologically diagnosed case by angiography was reported by Siedel in 1965 [12]. The variability in origin of the vessels that normally arise from external carotid artery may be explained in embriyologic terms [9]. Lasjaunias and colleagues reported that occipital artery is formed from proatlantal artery [14]. In early gestation, the proatlantal artery anastomoses with primitive internal carotid, external carotid and vertebral arteries. Anastomotic vessel between the primitive internal carotid artery usually regresses. Persistence of this anastomotic vessel and regression of the most proximal segment of the proatlantal artery result in the formation of an occipital artery of internal carotid origin. The incidence of anomalously originating occipital artery from the internal carotid artery is reported as 0.14% and 0.2% in two different studies performed by MRA and 3D CTA, respectively [2,3].

To our knowledge, there are only two reported cases in the literature with bilateral occipital arteries of internal carotid origin [3,5]. High grade stenosis of the left vertebral artery resulting in collateral flow to the vertebral artery via the muscular branches of the occipital artery of internal carotid artery origin is the unique feature of this third reported case.

Anomaly of internal carotid artery originating occipital artery is commonly asymtomatic [2], however, rarely it may have clinical importance in patients with carotid bifurcation and/or proximal vertebral artery obstructive lesions. Newton et al. reported an asymptomatic case of proximal internal carotid artery occlusion [13]. Collaterals between the vertebral artery and midportion of the internal carotid artery through an anomalously originating occipital artery provided the blood flow in this case. Anomalous occipital artery can be a source of collateral flow from the proximal internal carotid artery to the distal vertebral artery in stenosis of the proximal vertebral artery as presented in this case. In the endovascular treatment of patients with carotid artery stenosis, occipital artery with anomalous origin from the internal carotid artery must be regarded if there is collateral flow with an accompanying vertebral artery stenosis. Stenting of the carotid bifurcation stenosis may diminish blood flow to the posterior circulation via occipital artery to vertebral artery anastomosis and may result in ischemia. Redistribution of the atherosclerotic plaque material during baloon dilatation and/or stenting of carotid bifurcation may obstruct external carotid artery orifice or an anomalous branch of internal carotid artery such as occipital artery. Anomalously branching occipital arteries may also cause misdiagnosis during radiologic examinations. In a reported case, whose occipital artery arising from the stump of internal carotid artery simulated continuation of the internal carotid artery and was misdiagnosed as a stenotic segment in Doppler ultrasonography [15]. Selective angiography actually revealed the occlusion of the internal carotid artery. Accompanying atherosclerotic plaques of the carotid arteries and vertebral artery severe stenosis may effect the clinical importance and endovascular treatment risk in such an extremely rare variation.

## Competing Interests

The authors declare that they have no competing interests.
